# Primary 21-Gene Recurrence Score and Disease Outcome in Loco-Regional and Distant Recurrent Breast Cancer Patients

**DOI:** 10.3389/fonc.2020.01315

**Published:** 2020-07-31

**Authors:** Yujie Lu, Yiwei Tong, Jiahui Huang, Lin Lin, Jiayi Wu, Xiaochun Fei, Ou Huang, Jianrong He, Li Zhu, Weiguo Chen, Yafen Li, Xiaosong Chen, Kunwei Shen

**Affiliations:** ^1^Department of General Surgery, Comprehensive Breast Health Center, Ruijin Hospital, Shanghai Jiao Tong University School of Medicine, Shanghai, China; ^2^Department of Clinical Laboratory, Ruijin Hospital, Shanghai Jiaotong University School of Medicine, Shanghai, China; ^3^Department of Pathology, Ruijin Hospital, Shanghai Jiao Tong University School of Medicine, Shanghai, China

**Keywords:** breast cancer, 21-gene RS, recurrence, prognosis, first-line treatment

## Abstract

**Background:** The 21-gene recurrence score (RS) assay has been proven prognostic and predictive for hormone receptor-positive/HER2-negative, node-negative early breast cancer patients. However, whether primary 21-gene RS can predict prognosis in recurrent breast cancer patients remained unknown.

**Patients and Methods:** Consecutive breast cancer patients operated in Comprehensive Breast Health Center, Shanghai Ruijin Hospital between January 2009 and December 2018 were retrospectively analyzed. Patients with available 21-gene RS result for the primary tumor and reporting disease recurrence during follow-up were included. Association of 21-gene RS and overall survival (OS), post-recurrence overall survival (PR-OS), post-recurrence progression-free survival (PR-PFS), and first-line systemic treatment after recurrence were compared among different groups.

**Results:** A total of 74 recurrent patients were included, with 10, 27, 37 patients in the RS <18, 18–30, and ≥ 31 groups, respectively. Recurrent patients with RS ≥ 31 were more likely to receive chemotherapy as their first-line treatment compared to those with RS <31 (*P* = 0.025). Compared to those with RS <31, patients with RS ≥ 31 had significantly worse OS (*P* = 0.025), worse PR-OS (*P* = 0.026), and a trend of inferior PR-PFS (*P* = 0.106). Multivariate analysis demonstrated that primary ER expression level (OS: *P* = 0.009; PR-OS: *P* = 0.017) and histological grade (OS: *P* = 0.003; PR-OS: *P* = 0.009), but not primary 21-gene RS (OS: *P* = 0.706; PR-OS: *P* = 0.120), were independently associated with worse OS and PR-OS.

**Conclusions:** High primary 21-gene RS tended to be associated with worse disease outcome in loco-regional and distant recurrent breast cancer patients, which could influence the first-line systemic treatment after relapse, warranting further clinical evaluation.

## Introduction

Breast cancer (BC) is the most common global malignancy in women (1, 2). Despite standard comprehensive treatment according to clinical and histopathological features, 20–30% early-stage BC will develop loco-regional recurrence (LRR) and/or distant metastasis (3, 4). Previous meta-analysis involving 13,785 patients from 11 trials declared that a majority part of distant metastatic BC patients died within 2 years after recurrence (5). However, disease outcome of recurrent patients is highly variable and hard to predict, especially in hormone receptor (HR)-positive patients (6).

There are plenty of studies that analyze the predictive and prognostic factors on disease progression in early BC patients (7). Risk of BC recurrence was shown to be related to axillary lymph node status (8), primary tumor size, and tumor grade (9, 10). However, there remained controversies in the metastatic setting (11, 12). Tobin et al. revealed that the molecular subtype of metastatic lesions had prognostic value on post-recurrence survival (PR-OS) (13). Other retrospective studies demonstrated that primary ER status, adjuvant therapy, recurrence free interval, and first recurrence location were independently associated with survival in metastatic BC patients (14–16). Defining predictive and prognostic factors in the metastatic setting offered great challenges as well as opportunities toward an improved management of BC patients.

Over the past two decades, several gene expression signatures by microarray analysis have been developed in effort to predict prognosis and chemotherapy (CT) benefit (17). Among them, the 21-gene Recurrence Score (RS) assay was most widely applied and evaluated in clinical trials, which can predict both the benefit of adjuvant CT (18) and prognosis in HR-positive, human epidermal growth factor receptor-2 (HER2)-negative, node-negative patients (19, 20). The 2019 National Comprehensive Cancer Network Clinical Practice Guideline (21) also suggested to spare selective low-risk patients from adjuvant CT, while to apply adjuvant CT in high-risk patients based on RS results according to TAILORx and NSABP B-20 trials (22, 23). Nevertheless, the predictive or prognostic value of primary tumor 21-gene RS in recurrent BC patients has so far yet to be determined (24). Therefore, the aims of the current study are to evaluate the prognostic value of primary 21-gene RS for recurrent BC patients, as well as to identify whether 21-gene RS would influence subsequent first-line systemic treatment choice.

## Patients and Methods

### Study Population

Consecutive BC patients treated in the Comprehensive Breast Health Center, Shanghai Ruijin Hospital, Shanghai Jiao Tong University School of Medicine, Shanghai, China, were retrospectively included. Eligibility criteria were as follows: (1) patients receiving surgery between January 2009 and December 2018; (2) with available 21-gene RS results on primary tumor; (3) with complete clinico-pathological characteristics and immunohistochemical results for primary tumor; (4) reporting disease recurrence during follow-up period. Detailed data were retrieved from Shanghai Jiao Tong University Breast Cancer Database (SJTU-BCDB). Current study was approved by the independent Ethical Committees of Ruijin Hospital, Shanghai Jiao Tong University School of Medicine. All procedures were in accordance with the 1964 Helsinki declaration and its later amendments.

### Histo-Pathological Analysis

At least two experienced pathologists from the Department of Pathology, Ruijin Hospital, Shanghai Jiao Tong University School of Medicine, contributed to the tumor histo-pathological analysis. Positive criteria for IHC assessment of estrogen receptor (ER), progesterone receptor (PR), HER2, and Ki67 were as described in our previous reports (25). ER ≥ 50% was classified as high-expression (26). Molecular subtype was classified according to the 2013 St. Gallen expert panel consensus. Luminal A subtype was defined as ER+/PR ≥20%/HER2-/Ki-67 <14%, while Luminal B subtype was defined as ER+/HER2-/Ki-67 ≥14%, or ER+/PR <20%/HER2-, or ER–/PR+/HER2-.

### 21-Gene RS Assay Testing

The examination of the 21-gene RS assay was as reported in our previous work (23). Unstained breast tumor formalin fixed paraffin-embedded (FFPE) sections, from which RNA was extracted, were carefully selected by experienced pathologists in the Department of Pathology to ensure that tumor tissue consisted of at least 50% of the section. RNeasy FFPE RNA kit (Qiagen, 73504, Germany) and Omniscript RT kit (Qiagen, 205111, Germany) were applied in RNA extraction and reverse transcription process. Quantitative real-time polymerase chain reaction (RT-PCR) was conducted in Applied Biosystems 7500 Real-Time PCR System (Foster City, CA) using Premix Ex TaqTM (TaKaRa Bio, RR390A). The expression of genes was confirmed in triplicate, and normalized according to five reference genes *Beta-actin, GAPDH, RPLPO, GUS*, and *TFRC*. Regarding each gene expression level, patients were divided into two groups by using the median gene expression level as the cutoff value.

### Follow-Up

Patient follow-up was accomplished by specialized BC nurses or staff in our center. Disease recurrence included ipsilateral and loco-regional, distant metastasis in any site, contralateral invasive BC, and death of any cause. Overall survival (OS) was calculated from the date of surgery to death of any cause. PR-OS was computed from the date of first proven disease recurrence to death of any cause. Post-recurrence progression-free survival (PR-PFS) was estimated from the date of first proven recurrence till the date of first-line disease progression. Last follow-up was conducted in November 2019.

### Statistical Analysis

Patients were classified into two risk groups: low/intermediate-risk RS (RS <31) and high-risk RS (RS ≥ 31). Categorical data were analyzed using Chi-square test. Kaplan-Meier curve was conducted to compare OS, PR-OS, and PR-PFS differences between two RS groups. Cox regression was applied to identify influence factors for disease outcome. SPSS Version 23.0 (IBM Corp. Released 2015. IBM SPSS Statistics for Windows, Version 23.0. Armonk, NY: IBM Corp. from https://www.ibm.com/support/pages/how-cite-ibm-spss-statistics-or-earlier-versions-spss) and GraphPad Prism version 7.0 (GraphPad Software, CA, USA) were used in the analysis and data interpretation. Two-sided *P* < 0.05 was considered statistically significant.

## Results

### Patient Baseline Characteristics

**Figure 1:**
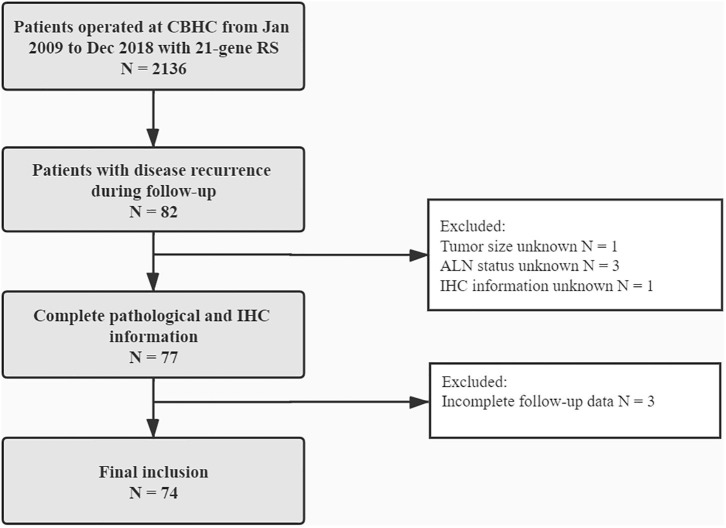
Flowchart of included patients. CBHC, Comprehensive Breast Health Center; RS, recurrence score; ALN, axillary lymph node; IHC, immunohistochemical.

In all, 2,136 BC patients with 21-gene RS records were retrospectively reviewed, and their characteristics were listed in Supplementary Table S1. Eighty-two patients reported disease recurrence during follow-up, including 20 LRR and 62 distant metastases. Patients with incomplete pathological, or follow-up information were then excluded. Overall, 74 patients were enrolled for final analysis (Figure 1). Nineteen patients (**Table 2**) reported LRR alone, 55 patients had distant metastasis with or without LRR.

Detailed clinico-pathological features were presented in Table 1. Median age was 53.0 (range 24–84) years. Forty-five patients were post-menopausal. Regarding primary surgery, mastectomy was performed in 44 patients and 37 patients received axillary lymph node dissection. Thirty-six patients had tumor larger than 2.0 cm, while 79.73% patients (*N* = 59) were node-negative. Sixty patients were diagnosed as invasive ductal carcinoma and grade 3 tumors were observed in 15 patients. Fifty-eight patients had ER ≥ 50%. Thirty-seven patients had PR <20%, of whom 17 were PR-negative. Luminal-B-like subtypes accounted for 87.84% (*N* = 65) of the whole study population. According to 21-gene RS, 10 (12.99%), 27 (37.66%), and 37 (49.35%) patients were categorized into low, intermediate, and high-risk groups, respectively.

**Table 1:** Baseline clinico-pathological characteristics of enrolled population.

	***N***	**%**
**Age, years (median, range)**	53.0 (24–84)	
≤ 50 years	27	36.49
>50 years	47	63.51
**Menstrual status**
Pre-menopausal	29	39.19
Post-menopausal	45	60.81
**Breast surgery**
Mastectomy	44	59.46
BCS	30	40.54
**Axillary surgery**
SLNB	37	50.00
ALND	37	50.00
**Histological type**
IDC	60	81.08
Others	14	18.92
**Histological grade**
2	45	60.81
3	15	20.27
NA	14	18.92
**Tumor size**
≤ 2 cm	38	51.35
>2 cm	36	48.65
**ALN involvement**
Negative	59	79.73
Positive	15	20.27
**ER**
<50%	16	21.62
≥50%	58	78.38
**PR**
<20%	37	50.00
≥20%	37	50.00
**Ki67**
<14%	24	32.43
≥14%	50	67.57
**Molecular subtype**
Luminal A	9	12.16
Luminal B	65	87.84
**21-gene RS**
<18	10	13.51
18–30	27	36.49
≥31	37	50.00

*BCS, breast conserving surgery; SLNB, sentinel lymph node biopsy; ALND, axillary lymph node dissection; IDC, invasive ductal carcinoma; NA, not available; ALN, axillary lymph node; ER, estrogen receptor; PR, progesterone receptor; RS, recurrence score*.

### Association of 21-Gene RS and First-Line Treatment Recommendation

**Figure 2:**
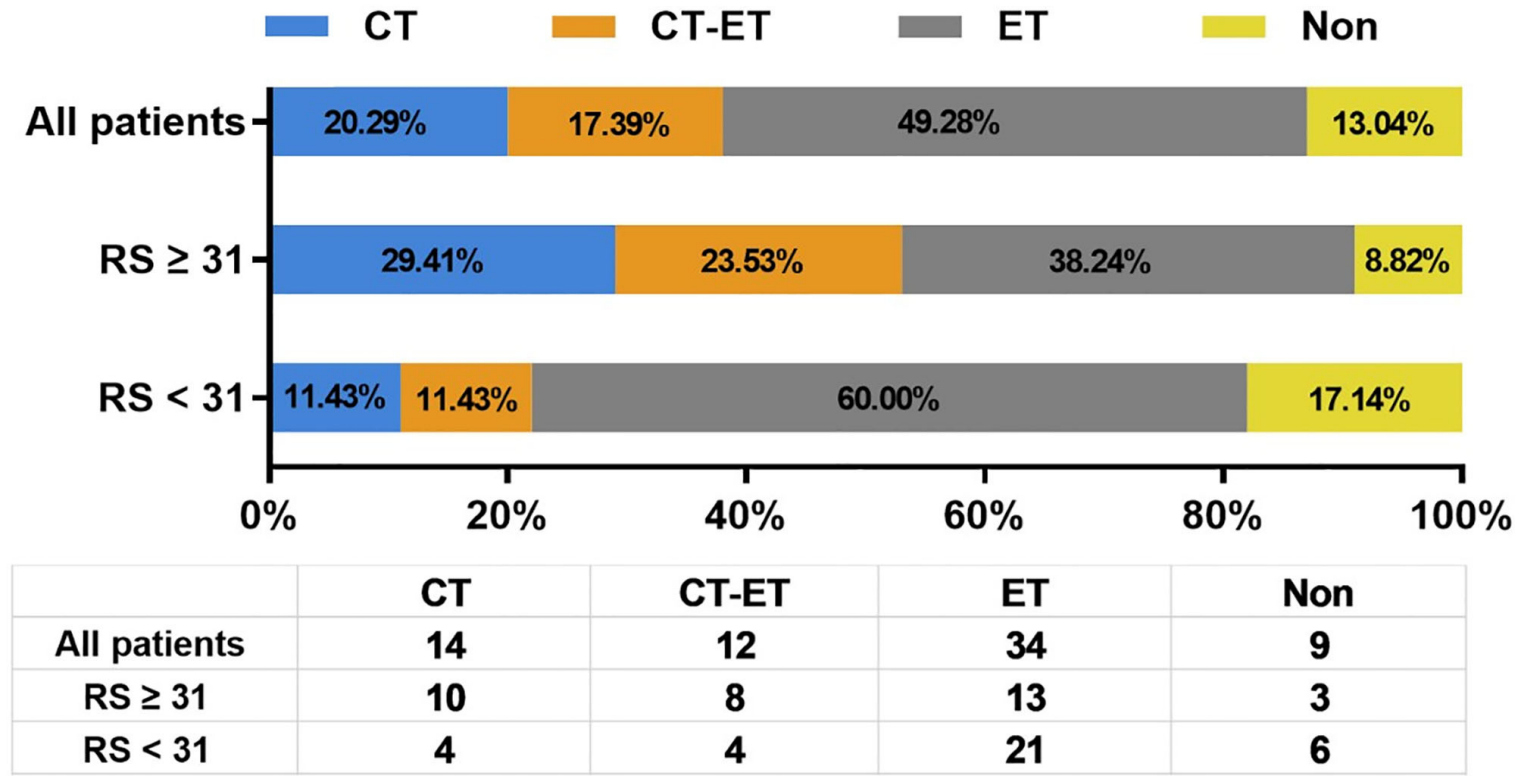
Distribution of first line systemic treatment recommendation after disease recurrence. RS, recurrence score; CT, chemotherapy; ET, endocrine therapy.

Figure 2 shows the first-line treatment selections after disease recurrence in patients with different RS. Therapy information was not available for 5 patients who received first-line treatments in their local centers. There were 20.29% (*N* = 14), 49.28% (*N* = 34), and 17.39% (*N* = 12) of enrolled patients received CT, endocrine therapy (ET), and CT followed by ET (CT-ET) as their first-line treatment, respectively. In addition, 6 patients with LRR alone or oligometastatic lesion underwent surgical resection without further systemic treatment. The rest 3 patients didn't receive further treatment due to elder age or other socio-economic factors.

As first-line systemic treatment in recurrent BC patients, CT, CT-ET, and ET were assigned to 10 (29.41%), 8 (23.53%), and 13 (38.24%) patients with RS ≥ 31, and 4 (11.43%), 4 (11.43%), and 21 (60.00%) patients with RS <31, respectively. Six (17.14%) low/intermediate-risk patients were spared from systemic treatment after recurrence. Compared to patients with primary RS <31, patients with primary RS ≥ 31 were more likely to receive CT as first-line treatment (*P* = 0.013).

When we adopted RS ≥ 26 as cutoff value to identify low/intermediate or high risk, details of first-line systemic treatment after relapse were described in Supplementary Figure S1. CT, CT-ET, and ET were assigned to 11 (24.44%), 9 (20.00%), and, 21 (46.67%) patients with primary RS ≥ 26, and 3 (12.50%), 3 (12.50%) and, 13 (54.17%) patients with RS <26, respectively. There was no statistically difference in first-line CT choice between primary RS ≥ 26 and RS <26 patients after recurrence (*P* = 0.127).

### Association of 21-Gene RS and Disease Outcome in Recurrent BC Patients

**Figure 3:**
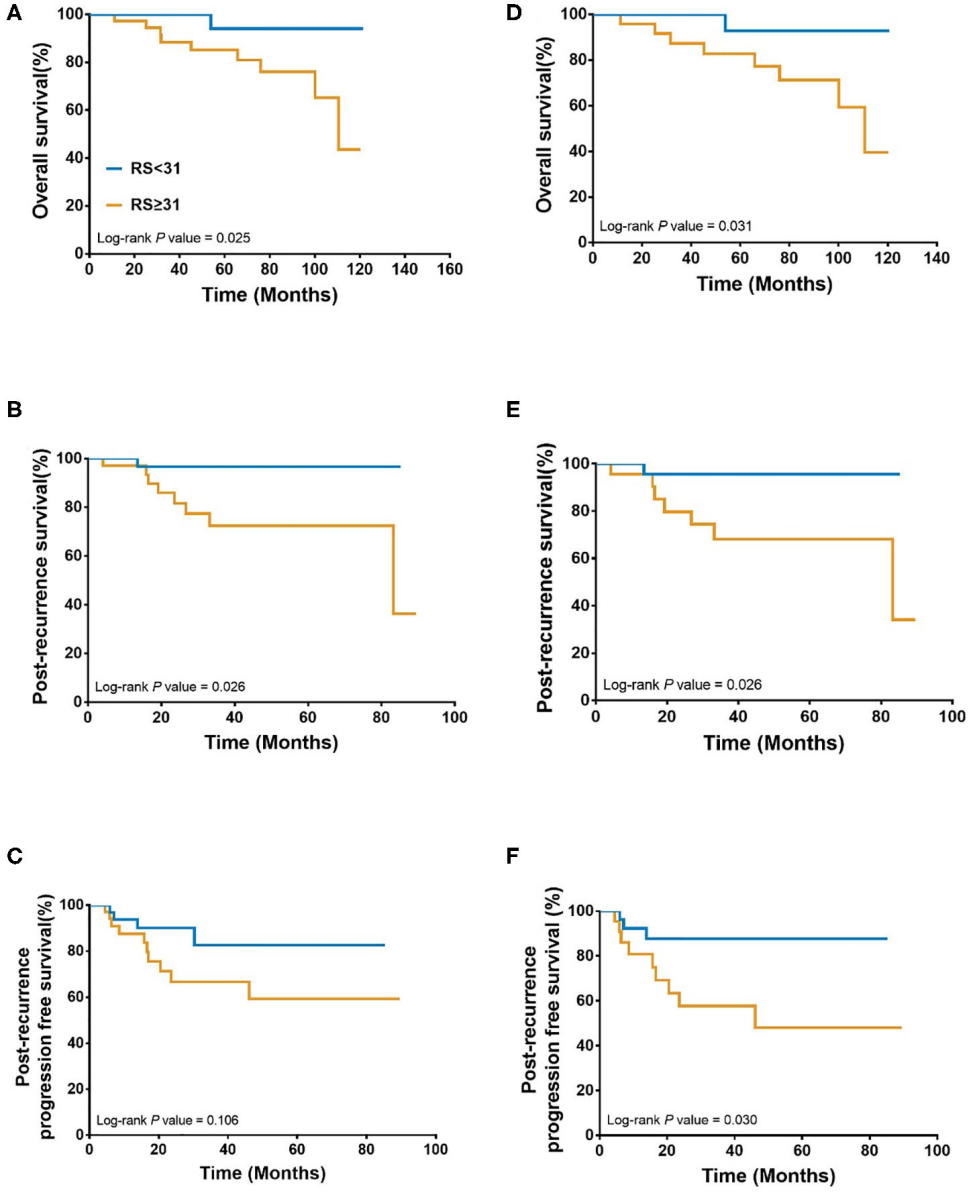
Association between 21-gene RS and survival in recurrent breast cancer patients. Overall survival **(A)**, Post recurrence survival **(B)**, and Post recurrence progression free survival **(C)** in the whole cohort; Overall survival **(D)**, Post recurrence survival **(E)**, and Post recurrence progression free survival **(F)** in patients with distant metastasis patients. RS, recurrence score.

Overall, during the follow-up of 2,136 patients with 21-gene RS, 82 patients had disease recurrence, 29 patients developed second primary malignancy, and 28 died (Supplementary Table S1). Among 74 patients finally included in our study with disease recurrence, the median follow-up was 57.90 months (range 7.60–121.50) and median post-recurrence follow-up was 25.70 months (range 0.37–89.47). Fifteen patients (Table 2) experienced disease progression after recurrence, in which 10 patients died. In patients with primary RS ≥ 31, 11 patients developed disease progression and 9 patients died. The rates of 5-year OS, 2-year PR-OS, and 2-year PR-PFS were 85.2, 72.5, and 66.7%, respectively. In patients with primary RS <31, 4 patients progressed and 1 patient died. The 5-year OS, 2-year PR-OS, and 2-year PR-PFS were 91.3, 81.6, and 90.1%, respectively. Patients with RS ≥ 31 had a significantly worse OS (*P* = 0.025, Figure 3A) and PR-OS (*P* = 0.026, Figure 3B) compared to patients with RS <31. In addition, histological grade (OS: *P* <0.001; PR-OS: *P* = 0.003, Table 3) and ER expression level (OS: *P* = 0.026; PR-OS: *P* = 0.008) were also related to OS and PR-OS in univariate model. PR expression level (*P* = 0.040), not RS categories (*P* = 0.106, Figure 3C), was associated with PR-PFS in univariate analysis.

**Table 2:** Disease recurrence and survival profile of enrolled patients.

	**All patients**	**RS <31**	**RS ≥ 31**
***N***	74	37	37
**Progression after recurrence**	15	4	11
**Death after recurrence**	10	1	9
**Recurrence sites**
Loco-regional recurrence	19	7	12
Distant metastasis	55	32	25
Visceral	21	13	8
Bone or soft tissues	30	17	13
CNS	4	0	4

*RS, recurrence score; OS, overall survival; PR-OS, post-recurrence survival; PR-PFS, post-recurrence progression free survival; CNS, central nervous system*.

**Table 3:** Univariate analysis of impact factors on OS, PR-OS, and PR-PFS.

**Clinico-pathological characteristics**	***P*****-value**		
	**OS**	**PR-OS**	**PR-PFS**
Age	0.390	0.395	0.191
Menstrual status	0.627	0.566	0.905
Histological type	0.099	0.106	0.061
Histological grade	**<0.001**	**0.003**	0.262
Tumor size	0.912	0.978	0.818
ALN involvement	0.708	0.592	0.636
ER	**0.026**	**0.008**	0.273
PR	0.114	0.055	**0.040**
Ki67	0.219	0.159	0.673
RS	**0.025**	**0.026**	0.106

*OS, overall survival; PR-OS, post-recurrence survival; PR-PFS, post-recurrence progression free survival; ALN, axillary lymph node; ER, estrogen receptor; PR, progesterone receptor; RS, recurrence score. Bold values are statistically significant*.

In multivariate Cox regression, primary ER expression level and histological grade independently influenced OS (Supplementary Table S2) and PR-OS (Supplementary Table S3). However, 21-gene RS categories was no longer an independent impact factor for OS (*P* = 0.706) or PR-OS (*P* = 0.120) after adjusting above factors. Primary ER high-expression was associated with better OS (hazard ratio [HR] = 0.11, 95% confidence interval [CI] 0.02–0.57, *P* = 0.009) and PR-OS (HR = 0.18, 95% CI 0.05–0.74, *P* = 0.017) in recurrent BC patients. Patients with histological grade 3 tumor had a significantly worse OS (HR = 4.20, 95%CI 1.65–10.65, *P* = 0.003) and PR-OS (HR = 7.08, 95%CI 1.64–30.63, *P* = 0.009).

When further stratified by recurrence site, LRR patients had similar clinical outcomes (OS: *P* = 0.439; PR-OS: *P* = 0.439; PR-PFS: *P* = 0.695) with different RS. Meantime, distant metastatic patients with RS ≥ 31 had a statistically significant worse OS (*P* = 0.031, Figure 3D), PR-OS (*P* = 0.026, Figure 3E) and PR-PFS (*P* = 0.030, Figure 3F) compared to those with RS <31.

In addition, if RS ≥ 26 was applied as cutoff, we observed that primary RS ≥ 26 was associated with a worse OS in univariate analysis (*P* = 0.048, Supplementary Figure 2A), but not with PR-OS (*P* = 0.182) or PR-PFS (*P* = 0.333). Primary RS ≥ 26 was no longer associated with OS in multivariate analysis (*P* = 0.552, Supplementary Table S4).

### Association of Single Gene Expression in 21-Gene RS and Disease Outcome in Recurrent BC Patients

Regarding each gene expression and survival, univariate analysis found that *PR* (*P* = 0.007, Table 4), *CD68* (*P* = 0.004), and *GSTM1* (*P* = 0.004) expression levels were significantly associated with OS. *GRB7* (*P* = 0.046), *PR* (*P* = 0.001), *CD68* (*P* = 0.003), and *GSTM1* (*P* = 0.001) expression levels were significantly associated with PR-OS. *GSTM1* (*P* = 0.040) was significantly associated with PR-PFS. After adjusting clinico-pathological factors, gene expression level of *CD68* maintained independent association to OS (High vs. low-expression, HR = 0.07, 95%CI 0.01–0.68, *P* = 0.022) and PR-OS (High vs. low-expression, HR = 0.09, 95%CI 0.01–0.79, *P* = 0.029) in recurrent BC patients.

**Table 4:** Univariate analysis of association between single gene expression in primary tumor and OS, PR-OS, and PR-PFS.

**Gene expression**	***P*****-value**		
	**OS**	**PR-OS**	**PR-PFS**
*GRB7*	0.122	0.046	0.648
*HER2*	0.100	0.109	0.998
*ER*	0.050	0.051	0.128
*PR*	**0.007**	**0.004**	0.086
*Bcl2*	0.208	0.353	0.244
*CEGP*	0.309	0.217	0.332
*CCNB1*	0.415	0.206	0.735
*Ki67*	0.765	0.966	0.610
*MYBL2*	0.188	0.257	0.519
*STK15*	0.930	0.675	0.493
*SURV*	0.192	0.107	0.769
*CTSL2*	0.510	0.176	0.962
*STMY3*	0.320	0.630	0.336
*CD68*	**0.004**	**0.003**	0.075
*GSTM1*	**0.004**	**0.001**	**0.040**
*BAG1*	0.553	0.377	0.554

*OS, overall survival; PR-OS, post-recurrence survival; PR-PFS, post-recurrence progression free survival. Bold values are statistically significant*.

## Discussion

In our current study, we included 74 patients from 2,136 consecutive patients with recurrent disease and found that patients with RS ≥ 31 had significantly worse OS and PR-OS and a trend of inferior PR-PFS compared with those with RS <31. In addition, recurrent patients with RS ≥ 31 were more likely to receive CT as their first-line systemic treatment compared to those with RS <31. Moreover, we found several genes in RS assay were associated with disease outcome and *CD68* was independently associated with OS and PR-OS in recurrent BC patients.

BC recurrence and metastasis are the very major reasons for poor prognosis, causing almost half of BC death within 5 year (27–29). Unlike in early BC, the widely-accepted impact factors on cancer patients' outcome, such as tumor size and lymph node status, showed no influence on survival in metastatic setting (7), which were confirmed in our study. The 21-gene RS is the most widely used multigene assay in early BC with predictive and prognostic value in HR+/HER2-, node-negative patients (19, 22), and it showed independently prognostic value for survival in ER+/HER2- *de novo* stage IV BC patients in a previous prospective study (11). However, its prognostic or predictive value in recurrent BC patients is still undetermined. Falato et al. estimated 21-gene RS and other multigene signatures in 187 recurrent BC patients using cDNA Affymetrix GPL10379 microarray data (24). They did not find any association between primary 21-gene RS and patient's survival in metastatic setting. While in our study, we applied qRT-PCR method to determine the gene expression and calculated RS, and found that primary high-risk RS patients had a significant worse OS and PR-OS in the whole population, and an inferior OS, PR-OS, and PR-PFS in patients with distant metastatic disease. Such different findings between studies may be attributed to different study population and different testing techniques. Moreover, Falato's study included HR- or HER2+ BC patients, which was not indication for 21-gene RS testing right now (30). Furthermore, we found that primary ER low-expression and histological grade 3 tumor were associated with worse OS and PR-OS in recurrent patients, which was in consistent with previous studies and may guide our further patient risk classification and treatment decision (29, 31, 32).

Systemic treatment decision for recurrent BC patients was largely based on biomarker status, number and sites of recurrence, and response of prior therapies (21, 33). For ER+/HER2- recurrent BC patients, ET is the preferred option, whereas CT is mainly assigned to patients with visceral crisis (21). The 21-gene RS is widely used to guide adjuvant treatment decision in early BC patients, however, its value in metastatic BC patients was limited. King et al. evaluated that in ER+/HER2- *de novo* stage IV BC patients, CT was more likely to be assigned to intermediate/high-risk RS patients as first-line systemic treatment and more with low-risk RS patients received first-line ET (11). Currently, there was no consensus on the optimal cutoff value applicable in metastatic setting. Here in this study we adopted both 31 and 26 as cutoffs, and found that primary 21-gene RS ≥ 31, but not RS ≥ 26 was associated with significantly increased CT usage after recurrence in ER+/HER2- patients. Herein, our finding suggested that primary 21-gene RS could influence first-line systemic treatment choice in the recurrent setting, and RS ≥ 31 might be the preferred cutoff for clinicians to decide first-line systemic treatment after disease recurrence, which warranted further validation. In addition, Asad et al. found that RS showed different predictive value of CT benefit in patients with different primary tumor size in adjuvant setting (34). In our study cohort, 6, 31, and 32 patients had a primary tumor of ≤ 1, 1–2, and > 2 cm. 21-gene RS could predict chemotherapy choice in 1–2 cm tumors (*P* = 0.032, Supplementary Table S5), but not in tumor ≤ 1 cm (*P* = 1.000), or > 2 cm (*P* = 0.433), suggesting that primary tumor size may influence predictive value of 21-gene RS on CT choice in metastatic setting. However, the sample size of each subgroup was relatively small, which warrants further exploration with larger cohort and longer follow-up time.

Previous studies have demonstrated that single gene expression in 21-gene RS panel was associated with survival in early BC patients. *GSTM1* (35, 36), *ER*, and *PR* (37) were associated with better outcome, while *CD68* (38) and *GRB7* (39) was related with worse prognosis. In our study, we found that *PR* and *GSTM1* was independently associated with superior disease outcome. However, in contrast, our study found that patients with high expression level of *GRB7* or *CD68* had a better disease outcome. The possible reasons were our study only included patients who had disease recurrence. In addition, molecular biomarkers and gene expression would change after disease recurrence (40–42). Two earlier prospective studies demonstrated that ER, PR, and HER2 status changed between primary and recurrent lesions in 12.6, 31.2, and 5.5% of patients, respectively (43, 44). Moreover, 21-gene RS retest on recurrent BC lesions is still doubtful, and we didn't know the true concordance rate of 21-gene RS between primary and recurrent BC, deserving further clinical evaluation.

In current study, we firstly evaluate the prognostic value of primary 21-gene RS categories on recurrent BC patients and its influence on first-line systemic treatment decision after recurrence. Several limitations still existed in this study. Firstly, as a retrospective analysis, selection bias in study population was unavoidable. Besides, the diagnosis of disease recurrence was based on radiographic examination or histo-pathological result. So only patients with “observable” or “evaluable” event could be included, which might lead to deviation in time and diagnosis of recurrence. In addition, it was difficult to distinguish a true ipsilateral local recurrence developed after breast conservation with a new primary tumor, which may result in potential bias. In our study cohort, 6 patients developed new tumors in the ipsilateral breast, and all of their new tumors occurred in the same quadrant of primary tumor, had similar histology to primary tumor, and thus were considered as disease recurrence. Further molecular analysis of clonal differences should be accomplished to better classify the patients (45). What's more, the sample size of the study was relatively small to conduct additional subgroup analysis. Last but not least, median post-recurrence follow-up time was relatively short to explore the subsequent systemic treatment and its influence on PR-OS.

In conclusion, our study demonstrated that primary 21-gene RS tended to be associated with worse disease outcome in loco-regional recurrent and distant metastatic patients. Several genes in 21-gene RS panel showed prognostic value for recurrent BC, which requires further evaluation. Primary 21-gene RS could influence the first-line systemic treatment after relapse, warranting clinical validation.

## Data Availability Statement

The raw data supporting the conclusions of this article will be made available by the authors, without undue reservation.

## Ethics Statement

The study involving human participants was reviewed and approved by the independent Ethical Committees of Ruijin Hospital, Shanghai Jiao Tong University School of Medicine. The patients/participants provided their written informed consent to participate in this study.

## Author Contributions

All authors listed have made a substantial, direct and intellectual contribution to the work, and approved it for publication.

## Conflict of Interest

The authors declare that the research was conducted in the absence of any commercial or financial relationships that could be construed as a potential conflict of interest.

## Supplementary Material

Supplementary Figure S1 Distribution of first line systemic treatment recommendation after disease recurrence when adopted RS ≥ 26 as cutoff value.

Supplementary Figure S2 Association between 21-gene RS and survival in recurrent breast cancer patients when adopted RS ≥ 26 as cutoff value. Overall survival **(A)**, Post recurrence survival **(B)**, and Post recurrence progression free survival **(C)** in the whole.
