# Supplementary material for: Primary 21-Gene Recurrence Score and Disease Outcome in Loco-Regional and Distant Recurrent Breast Cancer Patients
Source: Front Oncol. 2020 Jul 31;10:1315. doi: 10.3389/fonc.2020.01315 (PMC7412719; doi:10.3389/fonc.2020.01315)
Supplement: Supplementary file 1 [file Data_Sheet_1.docx]

**Supplementary Table S1 Clinico-pathological characteristics and clinical outcomes of HR-positive/HER2-negative breast cancer patients with primary 21-gene RS (N=2136)**

|  | N | % |
| --- | --- | --- |
| Age, years (median, range) | 57.0 (24 - 93) |  |
| ≤ 50 years | 691 | 32.35 |
| > 50 years | 1445 | 67.65 |
| Gender |  |  |
| Female | 2119 | 99.20 |
| Male | 17 | 0.80 |
| Menstrual status |  |  |
| Pre-menopausal | 744 | 35.11 |
| Post-menopausal | 1375 | 64.89 |
| Breast surgery |  |  |
| Mastectomy | 1220 | 57.12 |
| BCS | 916 | 42.88 |
| Axillary surgery |  |  |
| SLNB | 1563 | 73.17 |
| ALND | 556 | 26.03 |
| No axillary procedure | 17 | 0.80 |
| Histological type |  |  |
| IDC | 1830 | 85.67 |
| Others | 306 | 14.33 |
| Histological grade |  |  |
| 1 | 260 | 12.17 |
| 2 | 1325 | 62.03 |
| 3 | 415 | 19.43 |
| NA | 136 | 6.37 |
| Tumor size |  |  |
| ≤ 2cm | 1482 | 69.38 |
| > 2cm | 654 | 30.62 |
| ALN involvement |  |  |
| Negative | 1800 | 84.27 |
| Positive | 336 | 15.73 |
| ER |  |  |
| < 50% | 156 | 7.30 |
| ≥ 50% | 1980 | 92.70 |
| PR |  |  |
| < 20% | 651 | 30.48 |
| ≥ 20% | 1485 | 69.52 |
| Ki67 |  |  |
| < 14% | 992 | 46.44 |
| ≥ 14% | 1144 | 53.56 |
| Molecular subtype |  |  |
| Luminal A | 738 | 34.55 |
| Luminal B | 1398 | 65.45 |
| 21-gene RS |  |  |
| < 18 | 466 | 21.82 |
| 18-30 | 1110 | 51.97 |
| ≥ 31 | 560 | 26.22 |
| Adjuvant chemotherapy |  |  |
| Yes | 1077 | 50.42 |
| No | 1059 | 49.58 |
| Adjuvant radiotherapy |  |  |
| Yes | 1015 | 47.52 |
| No | 1121 | 52.48 |
| Adjuvant endocrine therapy |  |  |
| Yes | 2069 | 96.86 |
| No | 67 | 3.14 |
| Clinical outcome^*^ |  |  |
| Recurrence-free | 1997 | 93.49 |
| Loco-regional recurrence | 20 | 0.94 |
| Distant metastasis | 62 | 2.90 |
| Second primary malignancy | 29 | 1.36 |
| Death of any cause | 28 | 1.31 |

* Clinical outcome was reported according to the first recorded event during follow-up.

Abbreviations: HR, hormone receptor; HER2, human epidermal growth factor receptor 2; RS, recurrence score; BCS, breast conserving surgery; SLNB, sentinel lymph node biopsy; ALND, axillary lymph node dissection; IDC, invasive ductal carcinoma; NA, not available; ALN, axillary lymph node; ER, estrogen receptor; PR, progesterone receptor.

**Supplementary Table S2 Multivariate analysis of impact factors associated with overall survival**

|  | HR | 95%CI | *P* value |
| --- | --- | --- | --- |
| Histological grade |  |  | 0.003 |
| 2 | 1.00 |  |  |
| 3 | 4.20 | 1.65-10.65 |  |
| ER |  |  | 0.009 |
| < 50% | 1.00 |  |  |
| ≥ 50% | 0.11 | 0.02-0.57 |  |
| RS |  |  | 0.706 |
| < 31 | 1.00 |  |  |
| ≥ 31 | 1.58 | 0.15-16.80 |  |

Abbreviations: HR, hazard ratio; CI, confidence interval; ER, estrogen receptor; RS, recurrence score.

**Supplementary Table S3 Multivariate analysis of impact factors associated with post-recurrence survival**

|  | HR | 95%CI | *P* value |
| --- | --- | --- | --- |
| Histological grade |  |  | 0.009 |
| II | 1.00 |  |  |
| III | 7.08 | 1.64-30.63 |  |
| ER |  |  | 0.017 |
| < 50% | 1.00 |  |  |
| ≥ 50% | 0.18 | 0.05-0.74 |  |
| RS |  |  | 0.120 |
| < 31 | 1.00 |  |  |
| ≥ 31 | 5.35 | 0.65-44.35 |  |

Abbreviations: HR, hazard ratio; CI, confidence interval; ER, estrogen receptor; RS, recurrence score.

**Supplementary Table S4 Multivariate analysis of impact factors associated with overall survival (RS cutoff of 26)**

|  | HR | 95%CI | *P* value |
| --- | --- | --- | --- |
| Histological grade |  |  | 0.003 |
| 2 | 1.00 |  |  |
| 3 | 4.20 | 1.65-10.65 |  |
| ER |  |  | 0.009 |
| < 50% | 1.00 |  |  |
| ≥ 50% | 0.11 | 0.02-0.57 |  |
| RS |  |  | 0.552 |
| < 26 | 1.00 |  |  |
| ≥ 26 | 2.13 | 0.18-25.58 |  |

Abbreviations: HR, hazard ratio; CI, confidence interval; ER, estrogen receptor; RS, recurrence score.

**Supplementary Table S5 First-line chemotherapy choice after recurrence in patients with different primary tumor size**

| Primary tumor size | RS < 31 | |  | RS ≥ 31 | | *P* value |
| --- | --- | --- | --- | --- | --- | --- |
|  | CT | non-CT |  | CT | non-CT |  |
| ≤ 1cm | 1 | 2 |  | 2 | 1 | 1.000 |
| 1-2cm | 4 | 12 |  | 10 | 5 | **0.032** |
| > 2cm | 3 | 13 |  | 6 | 10 | 0.433 |

Abbreviations: RS, recurrence score; CT, chemotherapy.
