# Supplementary figures and images for: Primary 21-Gene Recurrence Score and Disease Outcome in Loco-Regional and Distant Recurrent Breast Cancer Patients
Source: Front Oncol. 2020 Jul 31;10:1315. doi: 10.3389/fonc.2020.01315 (PMC7412719; doi:10.3389/fonc.2020.01315)

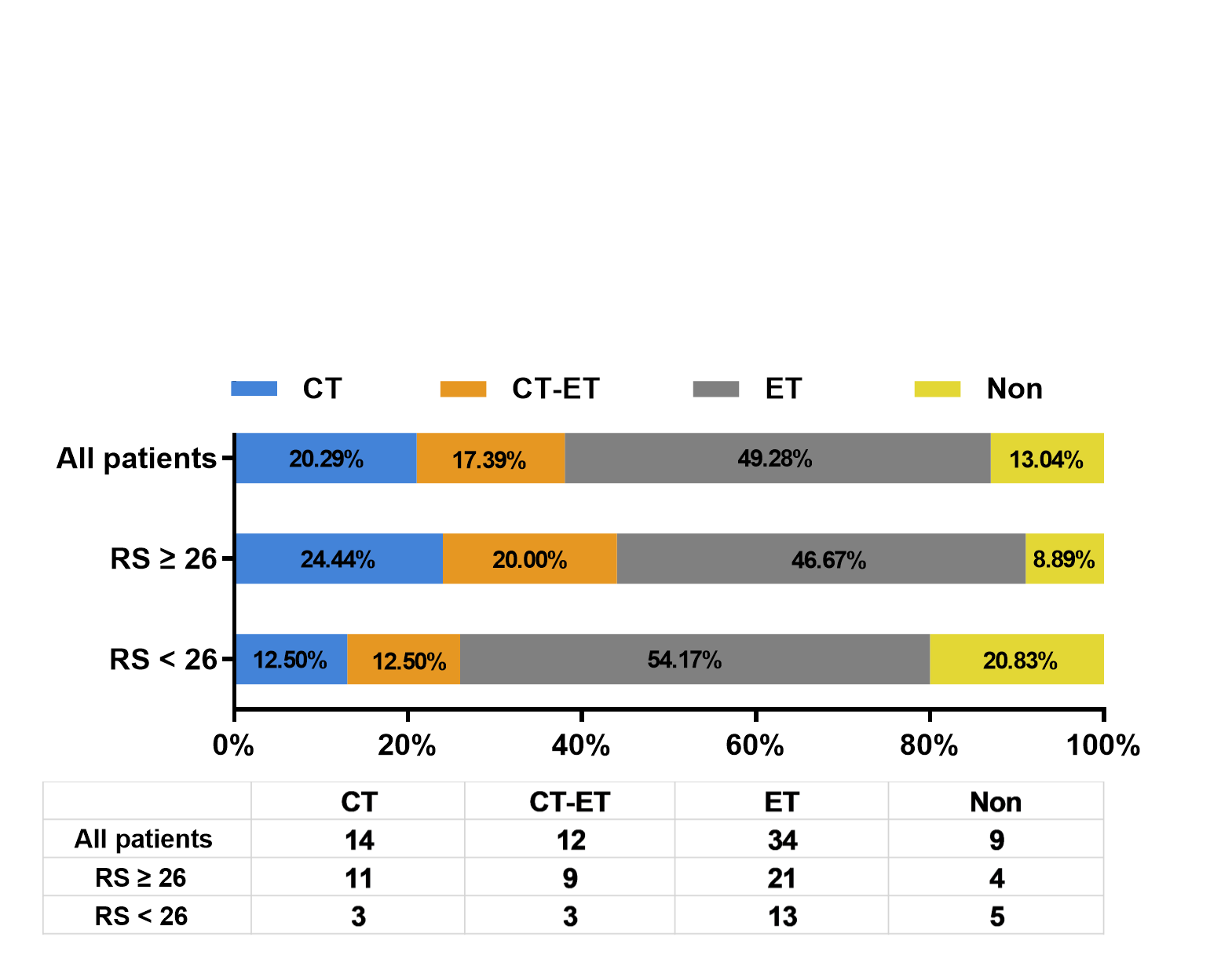

Supplement: Supplementary Figure S1 — Distribution of first line systemic treatment recommendation after disease recurrence when adopted RS ≥ 26 as cutoff value. [file Image_1.TIF]

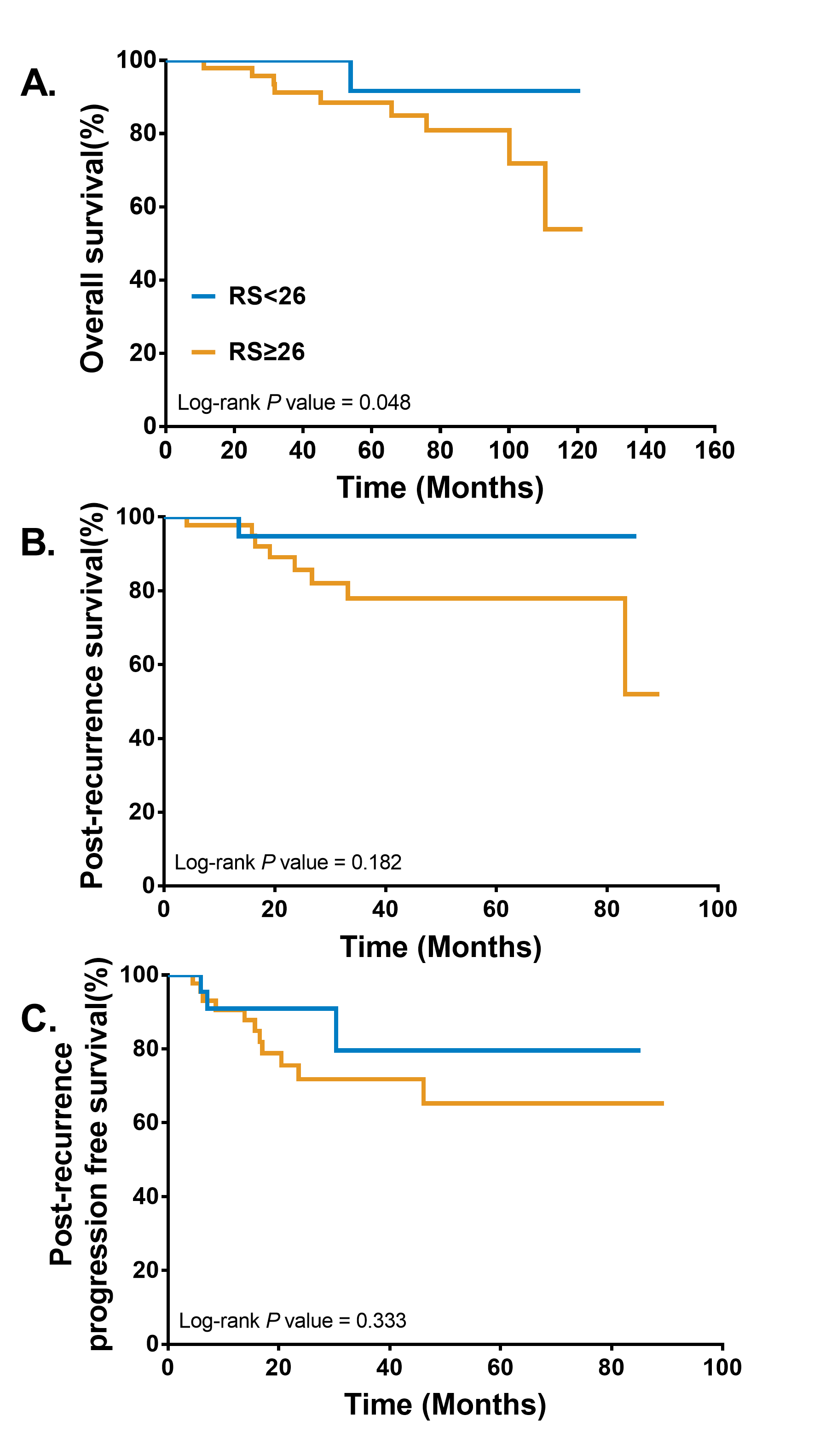

Supplement: Supplementary Figure S2 — Association between 21-gene RS and survival in recurrent breast cancer patients when adopted RS ≥ 26 as cutoff value. Overall survival (A), Post recurrence survival (B), and Post recurrence progression free survival (C) in the whole. [file Image_2.TIF]
